# From Aesthetic to Alarming: Ductal Carcinoma In Situ Following Breast Fat Grafting

**DOI:** 10.7759/cureus.85934

**Published:** 2025-06-13

**Authors:** Laila Ashkar, Bashair M Abufarea, Fai F Alkaeid, Raghad O Alruwaithi, Raghad W Nadhra

**Affiliations:** 1 Radiology, Faculty of Medicine, King Abdulaziz University, Jeddah, SAU; 2 Medicine and Surgery, Faculty of Medicine, King Abdulaziz University, Jeddah, SAU

**Keywords:** atypical ductal hyperplasia, case report, dcis, fat grafting, mammography

## Abstract

This case presents a rare occurrence of ductal carcinoma in situ (DCIS) in a patient with a history of bilateral iatrogenic fat injections, aiming to contribute to the ongoing discussion on potential associations between fat grafting and breast pathology. A 43-year-old Saudi woman presented with bilateral palpable masses at the sites of previous fat grafting performed two years earlier. Despite having no significant risk factors or family history of breast cancer, imaging revealed a suspicious asymmetry adjacent to the palpable area in the left breast. Biopsy of this region confirmed fat necrosis with foci of atypical ductal hyperplasia and low-grade DCIS. This case underscores the importance of vigilant follow-up in patients undergoing autologous fat grafting to the breast.

## Introduction

Autologous fat grafting is a surgical procedure commonly used for both reconstructive and aesthetic purposes. While it is generally considered safe, it can lead to several benign outcomes, such as fat necrosis. However, recent scientific publications have raised concerns about the potential role of fat injections in the development of atypical breast pathologies, including ductal carcinoma in situ (DCIS) [1].

DCIS is characterized by malignant cells confined within the milk ducts, which may progress to invasive carcinoma if left untreated [2]. Emerging evidence suggests that adipose tissue-derived stem cells and growth factors present in the injected fat may alter the breast’s natural microenvironment, potentially promoting the growth of premalignant cells such as those seen in DCIS. This hypothesis posits that these alterations may activate dormant or pre-malignant cells, particularly in individuals with underlying genetic mutations or hormone-related risk factors [3].

The histopathological basis of atypical hyperplasia involves abnormal cellular proliferations that, while not malignant, are considered atypical changes in breast architecture and are recognized as premalignant lesions [4]. One reported case involved a woman who developed two breast lumps 12 months after receiving a fat injection. Mammography showed displaced breast parenchyma with prominent microcalcifications [5].

The present case concerns a 43-year-old woman who developed bilateral breast lumps two years after undergoing bilateral fat grafting for breast augmentation. This report aims to contribute to the limited body of literature exploring the potential association between fat grafting and the development of breast atypia and/or DCIS.

## Case presentation

**Figure 1:**
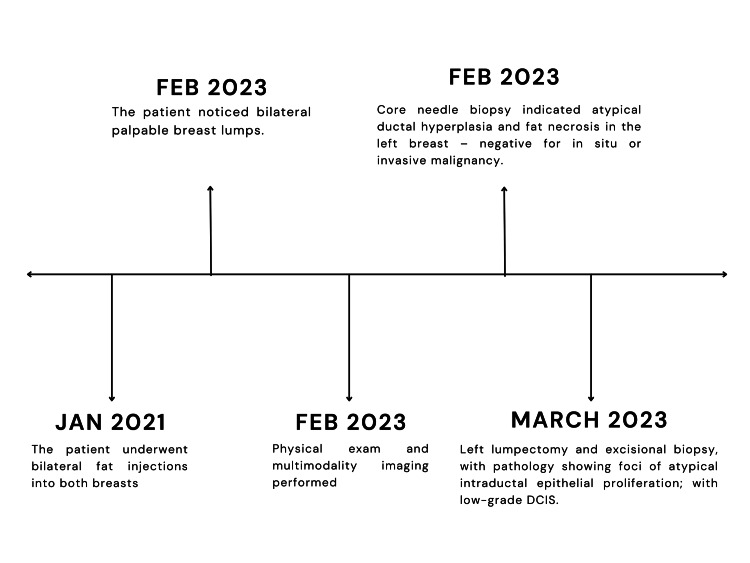
Timeline of case presentation Timeline illustrating the patient’s clinical progression following bilateral breast fat injections, including the development and evaluation of palpable breast masses. DCIS, ductal carcinoma in situ

A 43-year-old Saudi woman presented with bilateral palpable lumps in the central areas of both breasts, corresponding to the sites of fat injections performed two years earlier. She had previously undergone three rounds of IVF but had no personal or family history of breast or ovarian malignancies. On physical examination, her breasts appeared normal and symmetrical, with round, tender, palpable masses detected at the previous fat injection sites in both breasts. She reported no other breast symptoms, such as nipple discharge or skin changes, and no lymphadenopathy was noted (Figure 1).

**Figure 2:**
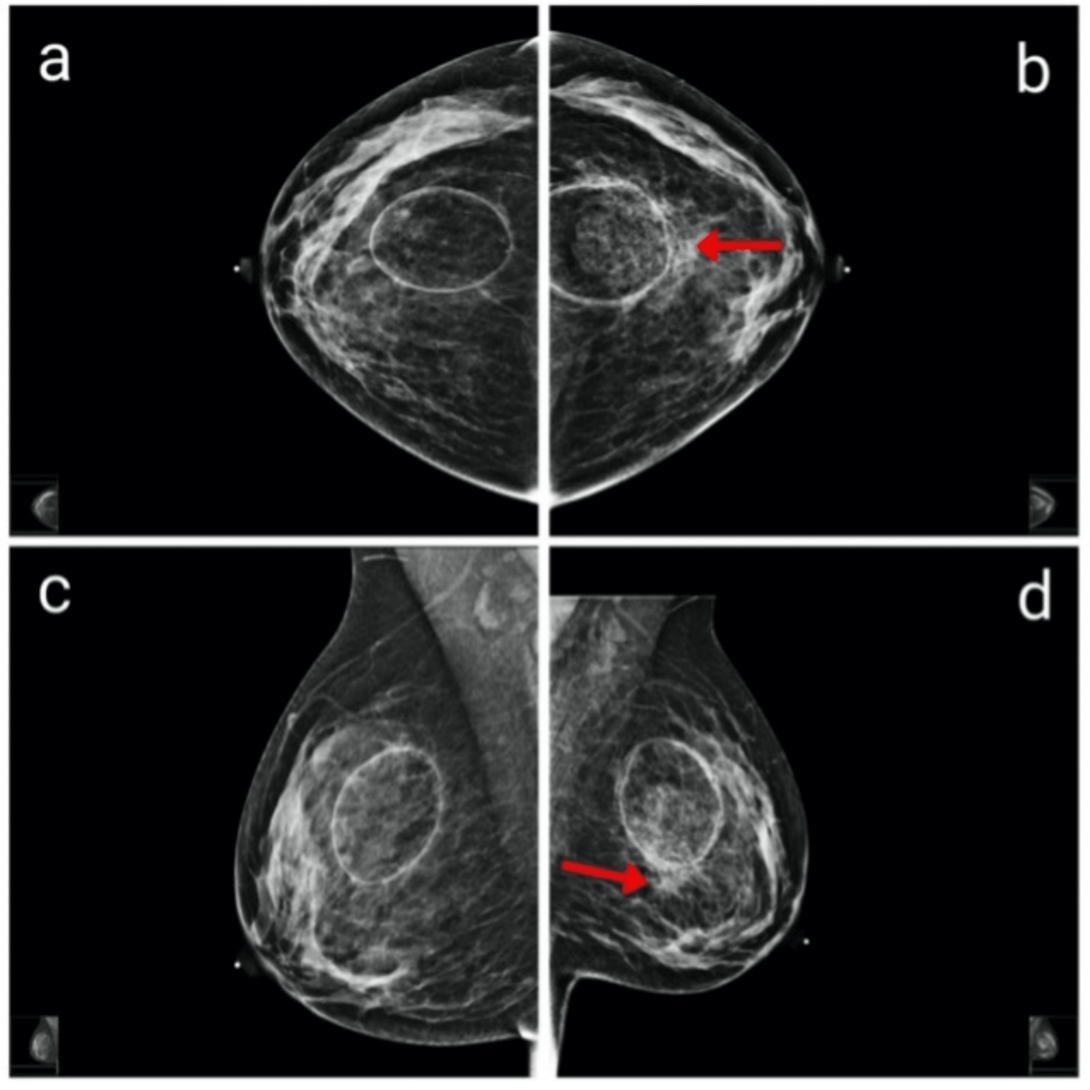
Pre-lumpectomy craniocaudal (a, b) and mediolateral oblique (c, d) mammograms Mammograms showing bilateral, well-circumscribed, fat-containing masses. In the left breast (b, d), an irregular focal asymmetry with architectural distortion is observed adjacent to the area of fat necrosis (see arrow).

Radiological assessment of the masses included breast ultrasound, bilateral mammography, and MRI. The radiologist found no malignant features in the right breast. Initial mammographic images showed heterogeneously dense breasts classified as ACR type C, which could obscure small masses (Figure 2). At the site of the palpable abnormality in the left breast, there was a large, oval-shaped, predominantly fatty mass at the 12:00 position, corresponding to the previous fat injection area, with rim calcifications. Within this fatty mass, a heterogeneous intrinsic mass with scattered calcifications was observed, along with an irregular spiculated mass at the anterior and inferior borders, causing architectural distortion. Similarly, in the right breast, a large oval, predominantly fatty mass was noted at the 12:00 position, also exhibiting rim and intrinsic calcifications.

**Figure 3:**
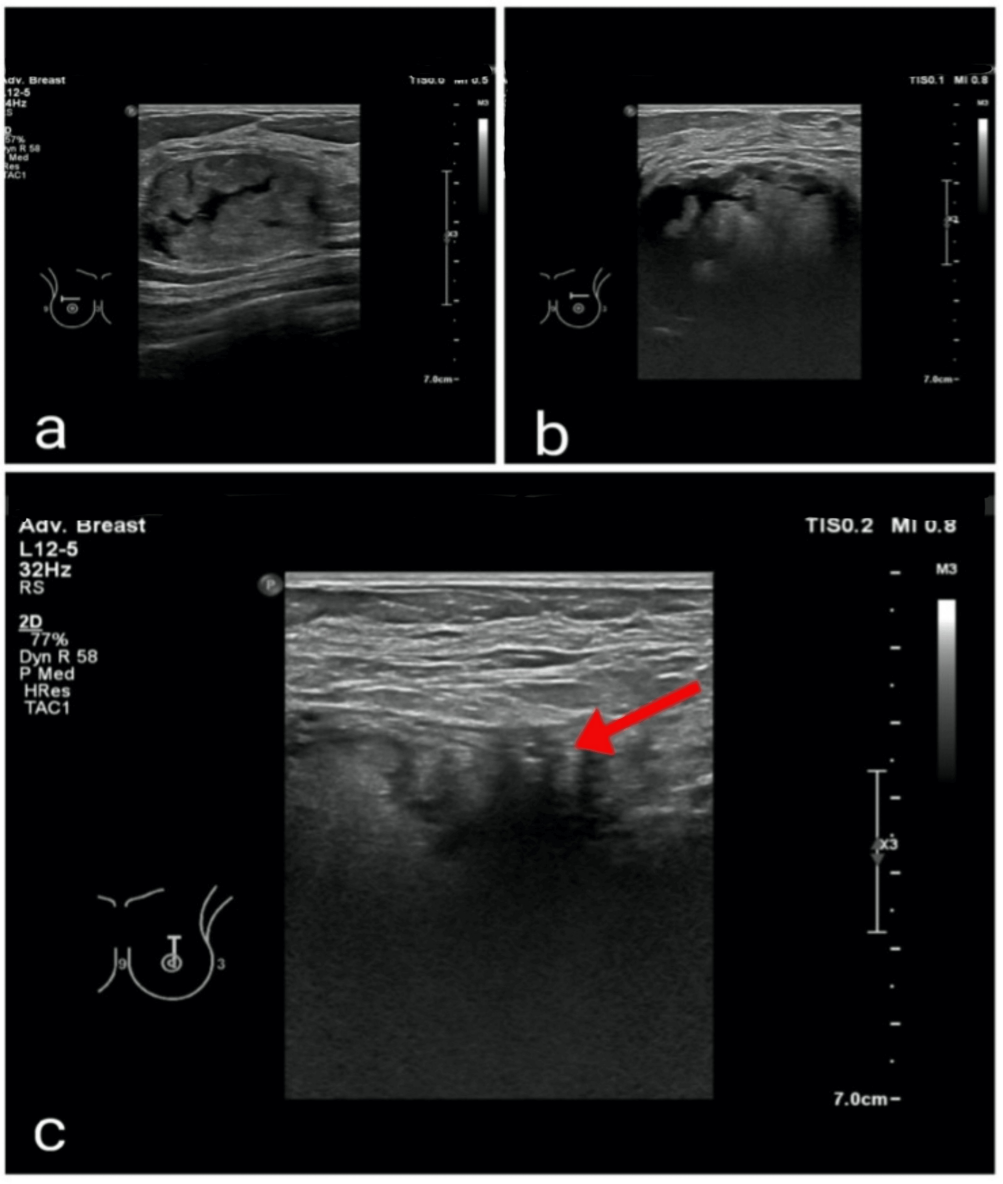
Pre-lumpectomy ultrasound Ultrasound images showing bilateral complex cystic and solid masses at the 12:00 position (a, b), with features suggestive of oil cysts or fat necrosis. In the left breast (c), an irregular mass with architectural distortion is observed adjacent to the area of fat necrosis (see arrow).

Additionally, bilateral prominent axillary lymph nodes with benign features were observed. Ultrasound of the left breast revealed a large, complex cystic and solid mass measuring approximately 5.1 × 3.6 × 5.1 cm at the 12:00 position, suggestive of an oil cyst or fat necrosis, with no increased internal vascularity (Figure 3). An irregular, spiculated hypoechoic mass with marked posterior shadowing was also identified. In the right breast, a corresponding complex cystic and solid mass measuring 4.0 × 2.7 × 6.1 cm was detected, consistent with oil cyst/fat necrosis and correlating with the palpable mass and the fatty mass observed on mammography.

**Figure 4:**
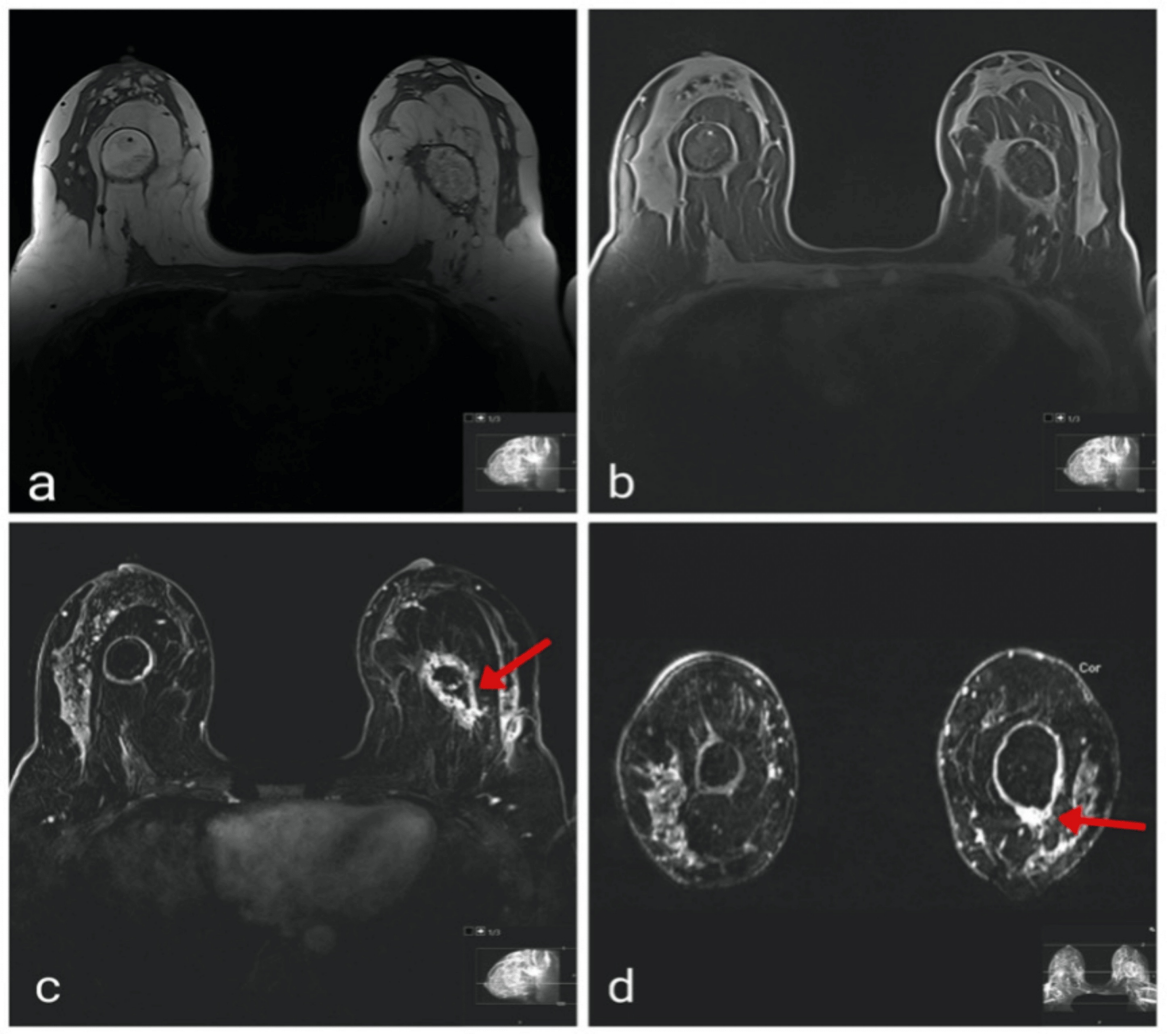
Pre-lumpectomy MRI T1 non-fat-saturated (a), T1 fat-saturated (b), axial (c), and coronal (d) subtraction MRI sequences showing bilateral oval, fat-containing masses. The left-sided lesion demonstrates a thick, enhancing wall. The red arrow indicates a suspicious, irregular, enhancing mass at the inferior margin of the fat necrosis in the left breast.

On MRI, multiple bilateral areas of peripheral nodular enhancement were observed, particularly in the inferior and lateral regions, corresponding to areas of fat necrosis (Figure 4). Both breasts showed oval fat-containing masses with rim calcifications that matched the large complex cystic and solid masses at the 12:00 position seen on ultrasound, suggesting fat necrosis from prior fat injections. The left breast mass exhibited a thick, enhancing nodular wall, with greater density and calcifications compared to the right side. An atypical irregular mass with architectural distortion and heterogeneous enhancement was identified in the inferior portion of the fatty left breast mass, raising suspicion and correlating with the spiculated mass noted on mammography.

**Figure 5:**
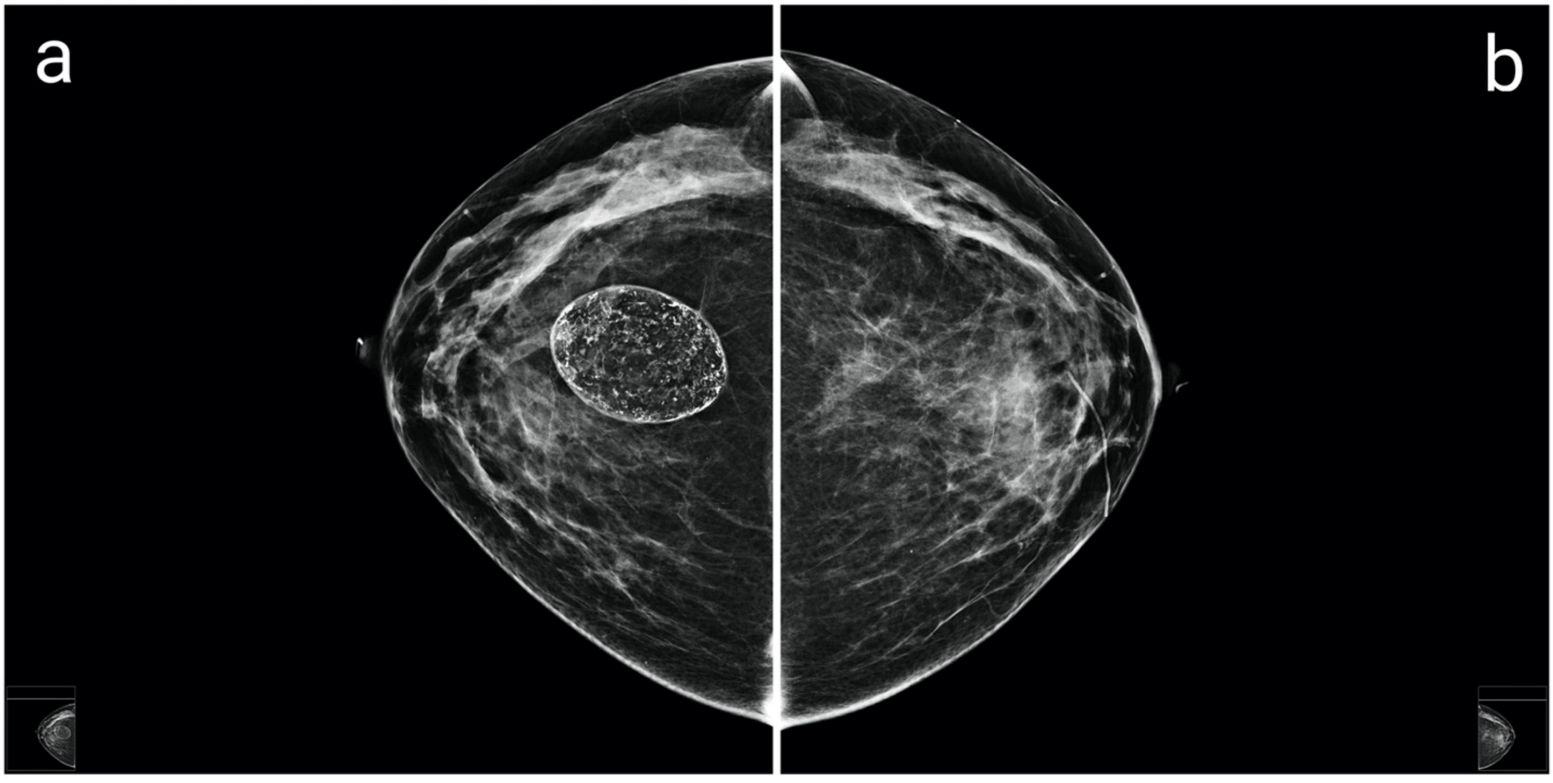
Post-lumpectomy mammogram (a) The right breast demonstrates increased rim and intrinsic calcifications within the oil cyst/fat necrosis, categorized as BI-RADS 2. (b) The left breast shows postsurgical architectural distortion following removal of the oil cyst/fat necrosis.

A biopsy of the left breast revealed atypical hyperplasia. Consequently, a lumpectomy was performed, and the excised specimen was reviewed alongside the previous core biopsy (pre-lumpectomy) (Figure 5). Both examinations showed foci of atypical intraductal epithelial proliferation, indicating that the lesion is quantitatively and qualitatively consistent with low-grade DCIS. While it is well established that fat injections can frequently lead to benign fat necrosis, the scientific relationship between these injections and atypical or malignant changes, particularly DCIS, has yet to be thoroughly studied.

Post-lumpectomy, the patient was advised to attend close follow-up appointments with mammography and ultrasound every six months. She was started on tamoxifen. Preventative recommendations also included lifestyle modifications and avoidance of factors that could trigger changes in breast tissue. Given her recent history of IVF, endocrinological follow-up was recommended to assess her risk and any hormonal effects on breast abnormalities. The patient remained under regular follow-up to monitor for stability or the development of any new breast changes. At her most recent visit, no new clinical findings were observed.

## Discussion

This case is among the few reports in the literature exploring the relationship between fat injections and atypical breast pathologies, including DCIS. Lipomodeling, also known as fat grafting, is commonly associated with benign outcomes such as fat necrosis, which can sometimes mimic malignancy upon clinical examination [3]. Research shows that the vast majority of masses developing after fat injection are benign; however, rare cases of atypical hyperplasia have been reported [1,3]. It is well established that approximately 30% of atypical hyperplasia cases may progress to DCIS, underscoring the importance of follow-up and long-term monitoring [4].

In this case, atypical hyperplasia was detected without evidence of invasive malignancy. This raises important questions about whether adipose tissue injection might alter normal breast architecture or provoke inflammation. This hypothesis aligns with the theory that adipose-derived stem cells and growth factors present in fat grafts could stimulate the ductal epithelium and immune response within breast tissue [2,5]. Such effects may vary depending on the injection technique, volume of fat transferred, and the inflammatory response elicited by the injected material. These considerations highlight the potential influence of fat grafting on breast cellular architecture [1,2].

Unfortunately, the literature lacks a clear consensus regarding the association between fat grafting and the development of atypia or malignant transformation. Experimental evidence from cell culture studies suggests that adipocytes may modify the local tissue microenvironment, potentially inducing atypical cellular behavior under certain conditions [5]. Although definitive confirmation is pending, some studies report that lipofilling might increase local recurrence risk in patients with prior intraepithelial lesions, while others discuss how stem cells within injected fat could affect adjacent epithelial cells and promote neoplastic transformation in specific contexts [6,7]. Therefore, this case supports the need for further research and long-term follow-up in patients undergoing fat grafting.

## Conclusions

The main takeaway from this case is the critical importance of clinical follow-up and long-term monitoring with imaging in patients receiving autologous fat injections. While fat necrosis generally results in benign findings, clinicians should maintain a high index of suspicion when confronted with atypical radiological features. This case emphasizes that, although the overall risk of malignancy is low, vigilance for DCIS or other atypical changes following fat grafting is warranted. As fat grafting procedures become increasingly common, this report highlights the necessity of postoperative surveillance to ensure early detection of concerning alterations in breast architecture.
